# Understanding consumer and clinician preferences and decision making for rehabilitation following arthroplasty in the private sector

**DOI:** 10.1186/s12913-017-2379-9

**Published:** 2017-06-19

**Authors:** Mark A. Buhagiar, Justine M. Naylor, Grahame Simpson, Ian A. Harris, Friedbert Kohler

**Affiliations:** 1 South West Sydney Clinical School, University of New South Wales, Elizabeth Drive, Liverpool Hospital, Liverpool, NSW 2170 Australia; 2 South West Sydney Local Health District, Locked Bag 7103, Liverpool, NSW 2170 Australia; 3 Whitlam Orthopaedic Research Centre, P.O Box 906, Caringbah, NSW 2229 Australia; 4 Ingham Institute of Applied Medical Research, PO Box 3151 Westfields Liverpool, Liverpool, NSW 2170 Australia; 5 HammondCare, 2/447 Kent St, Sydney, NSW 2000 Australia; 6 Level 2, 12 Victoria Rd, Parramatta, NSW 2150 Australia

**Keywords:** Consumer preference, Clinician preference, Joint arthroplasty, Rehabilitation

## Abstract

**Background:**

To understand private consumer and clinician preferences towards different rehabilitation modes following knee or hip arthroplasty, and identify factors which influence the chosen rehabilitation pathway.

**Methods:**

Mixed methods cross-sectional study involving 95 semi-structured interviews of consumers (patients and carers) and clinicians (arthroplasty surgeons, physiotherapists and rehabilitation physicians) in Sydney, Australia, during 2014–2015. Participants were asked about the acceptability of different modes of rehabilitation provision, and factors influencing their chosen rehabilitation pathway. Interviews were in person or via the telephone. Qualitative analysis software was used to electronically manage qualitative data. An analytical approach guided data analysis.

**Results:**

Pre-operative preferences strongly influenced the type of rehabilitation chosen by consumers. Key factors that influenced this were both intrinsic and extrinsic, including; the previous experience of self or known others, the perceived benefits of the chosen mode, a sense of entitlement, the role of orthopaedic surgeons and influence of patient preference, a patient’s clinical status post-surgery, the private hospital business model and insurance provider involvement. The acceptability of rehabilitation modes varied between clinician groups.

**Conclusions:**

No one rehabilitation mode provided following arthroplasty is singularly preferred by stakeholders. Factors other than the belief that a particular mode was more effective than another appear to dominate the pathway followed by private arthroplasty consumers, indicating evidence-based policies around rehabilitation provision may have limited appeal in the private sector.

**Electronic supplementary material:**

The online version of this article (doi:10.1186/s12913-017-2379-9) contains supplementary material, which is available to authorized users.

## Background

Over the past 15 years there has been a steady increase in the rates of primary total knee arthroplasty (TKA) and total hip arthroplasty (THA) procedures undertaken annually in Australia [1], reflecting trends also seen internationally [2–5]. These increasing rates of surgery have not only led to an increase in the utilisation of acute-care services, but also an increase in the utilisation of inpatient rehabilitation and other modes of post-operative therapy provision [5–7]. As the demand for these services has grown, the ongoing viability of the cost of inpatient rehabilitation in particular has been called into question [8]. A pilot investigation in Germany demonstrated that inpatient rehabilitation was not cost-effective when compared to an outpatient alternative following hip arthroplasty from the perspective of the healthcare insurer [9]. In order to restrain costs, policy changes in the US point towards tightening admission criteria restricting inpatient rehabilitation after arthroplasty surgery [10, 11]. Implicit in these changes is the assumption that arthroplasty patients can be managed by outpatient services in a more affordable manner, without compromising their healthcare outcomes.

In the context of TKA and THA no high-level evidence supporting or contesting the benefits of inpatient rehabilitation over outpatient or home programmes was available at the time this study was instigated. One Canadian randomised controlled trial (RCT) that combined hip and knee patients compared 18 days of inpatient rehabilitation to eight domiciliary sessions (physiotherapy in the home). No significant differences were shown in the outcomes measured, which included the Western Ontario and McMaster Universities Osteoarthritis Index (WOMAC), Short Form-36 and patient satisfaction [12].

In Australia, inpatient rehabilitation following arthroplasty surgery is most commonly utilised by private consumers i.e. those who are privately insured or elect to cover some or all of the costs of their healthcare, in contrast to public patients who have their healthcare needs met within the public health system, without payment. Recent estimates indicate that a median 40% of privately insured patients per surgeon were transferred to inpatient rehabilitation in 2014 following TKA, though this figure ranges from 0% to 100% [13]. This contrasts with the public sector utilization rate of 21% [14], suggesting that factors other than need drive the high utilization rate in the private sector. In the absence of conclusive evidence, the journey from the post-operative acute care setting to inpatient rehabilitation services is presumably guided by the consumers and clinicians involved, with various opportunities for the expression of preferences as decisions. While taking into account variances in the preferences of clinicians regarding rehabilitation alternatives [15], decisions around rehabilitation types utilised after arthroplasty are likely to be multi-dimensional. They may be guided by reasons related to a patient’s clinical status, but other factors, such as patient expectations, healthcare professionals’ personal preferences and related conveniences may also play a part [8, 16].

## Primary objectives

Given that there are serious questions about the long-term sustainability and cost effectiveness of broad and untargeted inpatient rehabilitation provision following arthroplasty surgery, a greater understanding of factors shaping consumer and clinician preferences, as well as the manner in which decisions relating to rehabilitation following surgery are made, will be invaluable when reviewing current services or designing new healthcare delivery systems [17]. Utilising a mixed methods design, the primary objectives of this study were to identify the preferences of private consumers (patient and carer) and clinicians [orthopaedic surgeon (OS), physiotherapist (PT), and rehabilitation specialist (RS)] for different modes of rehabilitation utilised after knee or hip arthroplasty and the factors which influence decision making for rehabilitation following surgery.

## Methods

### Recruitment and consent

All participants were volunteers and provided written, informed consent. Data collection occurred in two phases. Phase 1 involved consumers and was nested within a larger multicentre, observational study (ClinicalTrials.gov NCT01899443). Consecutive eligible private patients about to undergo arthroplasty surgery and their carers were invited to participate while attending a pre-operative admission clinic at one of two private arthroplasty hospital providers in New South Wales, Australia. Patient eligibility included having a principal diagnosis of osteoarthritis and was about to undergo either a unilateral or bilateral TKA or THA. Carer eligibility included being identified as the primary carer for one of these individuals. Sites with different business models were chosen, to investigate whether these may have an impact on a patient’s treatment after surgery.

Phase 2 involved key clinicians from three disciplines involved in the care of knee or hip arthroplasty recipients. A separate computer-generated randomisation list was created for each of the individual groups: orthopaedic surgeons, rehabilitation specialists and physiotherapists. For orthopaedic surgeons and physiotherapists, the hospitals listed in the Australian Orthopaedic Association National Joint Replacement Registry were used to generate a random sample of sites and a surgeon and physiotherapist at each identified site were invited to participate. For rehabilitation specialists and physiotherapists working in the sub-acute rehabilitation services, a list provided by the Australasian Rehabilitation Outcomes Centre was used to generate a random sample of sites, with the same method of recruitment and random sample generation utilised. All clinician participants had been responsible for the care of knee or hip arthroplasty recipients in the private healthcare sector in New South Wales within the last 12 months.

### Sampling and data collection

The determination of an appropriate sample size in qualitative research is a key component of the legitimacy of analysis and conclusions drawn [18]. Heterogenous sampling was used for both consumer and clinician components to capture a wide range of experiences relating to modes of rehabilitation [19]. The quality of the information collected was assessed after each interview, with consideration of newly emerging themes [20]. The sample ceased once it was determined that a sufficient number of information-rich cases had been drawn, coupled with saturation, i.e. no new information was being revealed from the interviews [21]. For patients, this occurred after 38 interviews, while the threshold was 19 for carers. It also took 19 interviews to reach this point with OS, while only 10 PT and eight RS interviews were required to reach this point due to the relative homogeneity of their responses.

Data for both consumers and clinicians were collected from one-to-one semi-structured interviews conducted between January 2014 and February 2015. For consumers, the semi-structured interviews were developed by clinicians familiar with the pre-admission and post-surgery settings, while for clinicians they were developed in consultation with an expert panel comprising an OS, RS and PT. Both were piloted before use. For patients, eight open-ended questions were posed which covered the following areas: the rehabilitation they had received, the process of decision-making they had undertaken, who had been involved in this decision, influencing factors, and options they had available (Additional file 1: Appendix 1). Four open-ended questions were asked of carers about their relationship with the patient and what they had done to support them post-surgery (Additional file 1: Appendix 2). There were seven open-ended questions posed to clinicians that canvassed their thoughts on inpatient rehabilitation and other options, as well as their current practices and options in this regard (Additional file 1: Appendix 3).

For consumers, demographic and other contextual data obtained at the time of consent included age, gender, and working status. In addition, Oxford Knee or Hip Scores [22] and EuroQol health related quality of life scores [23] were also obtained from patients. Consumer interviews took place approximately 6 weeks after the patient participants’ had their surgery. Data about clinicians included age, gender, years practised in the related field and, for OS, the number of lower limb arthroplasty’s performed annually.

In addition to the open-ended questions, five alternative rehabilitation types were presented in the interviews for participants to rate. These were based on modes provided in other countries and on those composed by the investigators based on current knowledge of patient preference for rehabilitation [24]. The options were: outpatient group therapy; outpatient one-to-one therapy, domiciliary therapy, hotel-based rehabilitation and inpatient rehabilitation. Consumers and carers were asked to rank the five options in order of preference. Clinicians were asked to rate the acceptability of each using a five point Likert scale, which was anchored with highly unacceptable and highly acceptable.

### Data analysis and management

The qualitative data were examined using principles of thematic analysis, a method utilised to identify, analyse and report patterns within data [25]. All interviews in this study were digitally recorded and transcribed verbatim and, as recommended by Miles et al. [26], were reviewed against audio recordings to maximise integrity and trustworthiness of data. This allowed for coding of ideas and understandings that may otherwise have been missed, and the ability to return to and recode old data. It also preserved the tone and tempo, silences and statements of participants [27].

Initial discursive codes were generated by one of the researchers (MB). The elucidation of these codes was assisted by a process of listening to and reading transcriptions of the audiotapes, as well as consulting colleagues and perusing relevant literature. QSR’s NVivo qualitative analysis software [28] was utilised to electronically manage data. An initial set of themes were categorised, examined for variability and consistency, re-checked against audio recordings and transcriptions, and then combined to outline the primary overarching factors and their components. These were then discussed with two researchers (JN and GS) to collate and refine the themes, so that clear, identifiable distinctions were developed between them [25]. In the final stages of analysis, earlier transcripts were re-read to ensure faithfulness of final results drawn, with emphasis placed on explaining the meaning and implications of each of the factors identified. Finally, categories were merged into larger groups, culminating in the finalisation of major and sub groups, and the drawing and verification of conclusions [26].

Descriptive statistics were generated for the demographic, clinical and quantitative components of the study.

## Results and interpretations

### Preferred rehabilitation modes/pathways

Respondent characteristics for consumer participants are listed in Table 1, and for clinician participants in Table 2. There were a variety of consumer-reported preferences for mode of rehabilitation provision post-surgery. Twenty five of 38 patients stated a preference for the same post-operative therapy they received if they were to have surgery again (Table 3). This trend was the same with carers, with 16 of the 19 carers nominating the mode of therapy delivery received by their spouse/parent as their preferred option. A variety of modes were selected as the second option for those who chose inpatient rehabilitation as their preferred option (Table 3). There was also variety in clinician-reported ratings of rehabilitation types (Table 4). One-to-one outpatient physiotherapy was the most highly rated by OS and PT, while inpatient rehabilitation was the most highly rated by RS.

**Table 1:** Consumer and carer respondent characteristics

Demographic	Consumers *n = 38*	Carers *n = 19*
Age (years), mean (sd)	66 (11)	63 (12)
Female, n (%)	25 (67)	11 (58)
Employed, n (%)	11 (29)	5 (26)
Oxford Score pre surgery, mean (sd)	23 (9)	-
EQ5D^a^ VAS^b^ pre surgery, mean (sd)	67 (17)	-
Knee surgery, n (%)	21 (55)	-
Unilateral surgery, n (%)	34 (89)	-
Previous arthroplasty, n (%)	7 (18)	-
Acute hospital length of stay (days), mean (sd)	6.2 (1.4)	-
Attended inpatient rehabilitation, n (%)	20 (53)	-

^a^EQ5D: EuroQol five dimensions questionnaire

^b^VAS: Visual Analogue Scale

**Table 2:** Clinician characteristics

Demographic	Orthopaedic surgeons	Rehabilitation specialists	Physical therapists
Age (years), mean (sd)	52.1 (6.7)	51.9 (7.8)	36.3 (13)
Female, n (%)	0 (0)	4 (50)	9 (90)
Years practising, mean (sd)	18.5 (7.1)	16.4 (12.9)	11.6 (10.2)
Operations per year, mean (sd)	161 (91)	-	-

**Table 3:** Rehabilitation preferences of patients

Considering mode received		
Demographic	Received (*n* = 38)	Prefer same mode
Supported home programme, n (%)	8 (21.1)	6 (75)
Outpatient based therapy, n (%)	6 (15.8)	3 (50)
Domiciliary therapy, n (%)	4 (10.5)	3 (75)
Inpatient therapy, n (%)	20 (52.6)	13 (65)
If inpatient rehabilitation was not available		
Demographic	Received (*n* = 15)	
Supported home programme, n (%)	0 (0)	
Outpatient based therapy, n (%)	2 (13.3)	
Domiciliary therapy, n (%)	5 (33.3)	
Hotel based therapy, n (%)	8 (53.3)

**Table 4:** Acceptability rating of rehabilitation modes by clinicians

Option	Orthopaedic surgeons	Rehabilitation specialists	Physical therapists
Outpatient rehab group	3.2	2.3	2.9
Outpatient 1:1 rehabilitation	4.1	3.5	4.6
Domiciliary rehabilitation	3.6	3.1	3.9
Hotel based rehabilitation	3.1	2.8	3.2
Inpatient rehabilitation	3.6	5.0	4.5

### Factors influencing the chosen rehabilitation mode/pathway

Two overarching factors and their components (Table 5) were identified, and are discussed in detail below.

**Table 5:** Factors influencing the chosen rehabilitation mode/pathway

Overarching factor types	Sub categories
Intrinsic: patient perceptions, experiences and beliefs	• Previous experience of self or known others • Perceived benefits • Sense of entitlement • Perceived unnecessary level of care/support
Extrinsic: clinician and service provider issues	• Role of orthopaedic surgeons, and influence of consumer preference for inpatient rehabilitation • Clinical status post-surgery • Private hospital business model • Insurance provider

### Intrinsic factors: Patient perceptions, experiences and beliefs

These factors related to the patient participants’ own perceptions of their situation, options and needs.

#### Previous experience of self or known other

Whether their own, or as reported by family and friends, a positive personal experience with inpatient rehabilitation was a common factor for patients who preferred this mode of rehabilitation delivery. Patients and carers frequently described the convenience of having everything at hand, from medical support to medication and meals:

*‘I had the medical service there if I needed it, I had pain relief on hand if I needed it, and it was a simple matter of walking down to the gym and hydro pool… twice a day and getting the regular exercise.’* (Patient 32)

*‘…just having the medical staff and the monitoring going on… so that she’s able to get fully mobile and gain the confidence to be able to get around, shower, and that sort of stuff’.* (Carer 5)

Patients also appreciated the opportunity to compare progress, and interact with others in the same situation as themselves:

*‘Being with the other patients and chatting and comparing notes was excellent too, ‘cause you felt that you weren’t alone*.’ (Patient 20)

However, in the same way that a personal positive experience with inpatient rehabilitation was a common factor for patients who preferred that mode of rehabilitation delivery, a personal positive experience with a mode other than inpatient rehabilitation often led patients and carers towards a preference for these modes. This highlighted the finding that patients and carers tended towards a preference for the same mode of therapy received if they were to have surgery again. This theme was also a factor for clinicians who rated modes other than inpatient rehabilitation highly, whether from the private or public health sector. As one OS stated:

*‘I think [outpatient rehabilitation] works very well for most [of] my public patients… they go straight home.’* (OS 7)

#### Perceived benefit of chosen rehabilitation mode

There was a strong sense from many of the consumers and clinicians that inpatient rehabilitation was an essential component of treatment. Several of the reasons for this perception revolved around clinical or social issues, and in some instances personal factors such as ‘laziness’ or a need for motivation. Factors frequently mentioned to have influenced a choice of inpatient rehabilitation included weakness, advanced age, the presence of significant comorbidities, the home environment, and a lack of support at home:

*‘[I recommend inpatient rehabilitation] if they’re old, if they’ve got significant comorbidities, if they lack family support, if they have more than 12 steps, if they’re slow while they’re in hospital.’* (OS 18)

*‘if they live alone, they are older, more deconditioned, they are slower, not motivated, or not able to comprehend what we are trying to get across to them as easily as some.’* (PT 2)

The RS interviewed also expounded the benefits of inpatient rehabilitation

*‘[Inpatient rehabilitation] can really improve the joint range of motion, pain management, confidence in managing mobility and functioning and preparing them better to be discharged home after rehabilitation.’* (RS 4)

The option of inpatient rehabilitation was also identified as an excellent option for people living in a rural environment.

*‘Inpatient rehab is a huge advantage because lots of our patients come from two, three hours away… and so by the time they drive [to the PT] and drive back, they’ve lost the benefit of the actual physio treatment session.’* (OS 13)

However, convenience was often a factor which led patients and carers towards a mode other than inpatient rehabilitation. The reasons for this perceived convenience were varied, from finding it easier to attend a private PT nearby (Patient 27) or having transport provided for outpatient therapy (Carer 19) to having a preference for the PT to come to one’s own home.

Competing priorities also played a part, particularly when patients had a role as a carer. For one woman (Patient 6) it was the needs of her daughter with a bi-polar disorder that led her to a preference for an early discharge with outpatient one-to-one therapy follow up, while for others it was the needs of a husband and household pets (Patient 5), or the prospect of a loss of income with a prolonged admission (Patient 38).

While outpatient services were well utilised, domiciliary physiotherapy was arranged in a number of cases, allowing patients to be treated in their own home. Others to whom it was not offered would have preferred it as an option if available:

*‘Staying away for a longer period of time is really hard for [my kids] and, yes, it would have been easier to have [the PT] actually coming to the house.’* (Patient 31)

Almost every patient who expressed a preference for a mode other than inpatient rehabilitation expressed that their preference was linked in some way to getting back to the familiar environment of their own home. For some it was simply being able to sleep in one’s own bed, others again spoke of the support of family, friends and neighbours. And while many patients and carers found the hospital environment of inpatient rehabilitation conducive to their recovery, that setting led others towards a preference for other modes.

#### Sense of entitlement

Consumers identified inpatient rehabilitation as a tangible way of getting value for the money they had put towards their private health insurance:

*‘…the fund was paying for [inpatient rehabilitation], so I was prepared to get the benefit of the whole thing’.* (Patient 10)

On some occasions this sense of entitlement ran alongside the perception that inpatient rehabilitation would also be advantageous for their recovery from surgery, but in others it seemed to be the primary motivating force for their preference. Clinicians verified this sense of entitlement, from OS:

*‘We do get a cohort of people who want their entitlement; they've paid for it…’* (OS 19)

to PT:

*‘…they’ve paid their private health insurance premiums for however many years, they think, “well, why shouldn’t I get to go [to inpatient rehabilitation], other people get to go. Yeah my knee might be good or my hip might be good but I want to go as well”.’* (PT 10)

In some instances, clinicians themselves shared these sentiments:

*‘I mean, to a large extent, they’re paying for private health insurance, so they’re entitled to go to [inpatient] rehab.*’ (OS 12)

#### Perceived unnecessary level of care/support

Consumers who expressed a preference for modes other than inpatient rehabilitation often spoke of it as unnecessary, given their individual circumstances. Some saw it as an option for ‘old’ people, while others referred to the support they had available at home as making an early discharge to that environment more advantageous to their recovery. For some, it was simply fondness for their own home environment, or dislike of the hospital environment, that led them in this direction.

When compared to those with a preference for inpatient rehabilitation, this group of consumers tended to describe a more supportive home environment, with services such as transport, cleaning and meal preparation more frequently available. Many patients interviewed indicated that they would have utilised transport, cleaning and meal services if these had been available. There was evidence of these being offered or available in some cases, and this having had an influence on decisions made. For example, when asked why inpatient rehabilitation was considered unnecessary, one carer responded:

*‘because [the patient] had transport provided so that he could stay here [at home] and just go and have his physio.’* (Carer 19)

A number of patients also indicated that they had utilised local community transport services for appointments.

### Extrinsic factors: clinician and service provider issues

There were varied experiences reported by patients following surgery, but most shared a number of common characteristics. Although the information provided at different sites varied, most patients reported attending a pre-admission visit to their nominated hospital, meeting with their surgeon prior to and after surgery, and having some physiotherapy intervention after discharge. However, there were a number of extrinsic factors both before and after surgery which led patients towards different modes of rehabilitation following arthroplasty. These factors were explored in the clinician interviews.

#### Role of orthopaedic surgeons, and influence of consumer preference for inpatient rehabilitation

As a group, OS reported differences of opinion regarding the perceived clinical value of inpatient rehabilitation following knee arthroplasty, and different approaches to recommendations made to their patients about post-operative care. For some surgeons, it was a case of one-size fits all:

‘*99% of my patients go to inpatient rehab… I tell them they’re going to go.’* (OS 14)

Others tailored the mode to the individual, with the default set as an alternative mode and inpatient rehabilitation only suggested if deemed clinically or socially advantageous:

*‘My words vary with every patient I see. My default position is that you go home and access outpatient facilities. However, if [they] are old, live alone, [or] have medical issues, then I understand that inpatient physio might be more appropriate’* (OS 3)

One surgeon, who expressed a conviction that inpatient rehabilitation had a positive impact on the clinical outcomes for his patients, also implied that this approach had the effect of attracting patients to his services:

*‘I think [inpatient rehabilitation] is a big part of what sells my joints to patients. I know surgeons who sent patients home one or two days after surgery [and] I get a lot of their patients.’* (OS 17)

However, while these variations in approach existed within the group, the impact of a consumer preference for inpatient rehabilitation on the decision made was consistently reported by all OS interviewed. All indicated that they would go along with a stated preference for inpatient rehabilitation, even if they didn’t see a clinical need for it:

*‘If the patient is really keen on it, I don’t say no. I don’t feel strongly about it, that they should or shouldn’t go.’* (OS 7)*;*

*‘…a patient will come and say “I want rehab post-op” and you think “Oh, you’re too fit and healthy, you don’t need that”, but if they want it, they get it.’* (OS 10)

This was even the case when it directly contravened the clinicians own personal preferences:

‘*In many occasions I’d prefer the patient to go home, but you’re caught between a rock and a hard place a lot of the time.’* (OS 12)

#### Clinical status post-surgery

The patient’s clinical status was reviewed in the acute phase of their recovery with OS and PT playing a part, and occasionally also nursing staff. Depending on their condition and rate of recovery at this point, any decision regarding path to rehabilitation type that had been made prior to surgery was either enacted, clarified or changed to take into consideration an unforeseen occurrence. The latter included post-operative complications, as well as better-than-expected patterns of recovery. RS reported playing virtually no part in this decision, which was confirmed by patients, OS and PT.

#### Private hospital business model

The journey towards or away from inpatient rehabilitation may have been influenced by the model of service delivery in place at the hospital in question. At one site, a number of patients interviewed went through surgery and outpatient therapy afterwards without even knowing that inpatient rehabilitation was an available option. Alternatively, at the second site, all patients interviewed were aware of inpatient rehabilitation as an option, and it was often presented as ‘part of the package’ at preadmission sessions. Although many patients indicated they had already made a decision in regards to their preferred mode of rehabilitation before attending these sessions, others were clearly influenced by this approach:

*‘I think it was just sort of a done deal that you went to rehab after.’* (Patient 9)

Clinicians confirmed this approach at other sites, and indicated that patients admitted for therapy to particular rehabilitation inpatient units may have had no significant clinical indicators for such therapy.

*‘…up here, the inpatient therapy, they can do their own thing… There is certainly a bit of encouragement … from the hospitals, because there is a financial benefit for them as well… The strike rate for people going to [inpatient] rehab [here] is almost one hundred percent, and I guess the reality is maybe 60% may not need it. Maybe they’ll do just as well at home. It’s just the reality.’* (OS 12)

*‘In our facility most of the patients are going straight into rehab. It’s like a normal routine.’* (PT 2)

A number of clinicians indicated that patients admitted for therapy to particular rehabilitation inpatient units may have had no significant clinical indicators for such therapy.

*‘There are people that come to rehab that don’t really need to come to rehab. It’s not really that anything bad happens because of it, just that they don’t really need to be here.’* (PT 5)

#### Role of private health funds

There were references to health funds through both consumer and clinician interviews, but in only one case was there evidence of direct involvement on the choice of mode of rehabilitation delivered. In that instance Patient 33 described how his health fund had arranged domiciliary therapy provision for him following surgery, as part of his coverage. The other references to health funds generally alluded to the fact that the funds would cover inpatient rehabilitation as a treatment option after surgery and, as detailed earlier, knowledge of this cover did lead to a sense of entitlement in some. However, other patients elected a mode other than inpatient rehabilitation even when aware they were covered for this option. When questioned, some patients were unclear of their entitlements in regards to health cover after surgery. One of the PTs commented on the accumulation of out-of-pocket expenses for patients who attended private outpatient therapy.

*‘It can get pretty expensive by the time they come in and pay the gap all the time.’* (PT 6)

## Discussion

Considering the concerns around the long-term sustainability and cost effectiveness of broad and untargeted inpatient rehabilitation provision following arthroplasty surgery, the findings of this study are timely, and provide a clear description of factors shaping consumer and clinician preferences, as well as how decisions relating to rehabilitation following surgery are decided upon.”

This study clearly exposes the many factors which influence private consumer and clinician preferences for particular modes of rehabilitation and associated decision-making following arthroplasty. These factors generally relate to clinical and social matters, but also include a sense of entitlement and extrinsic influences. The pattern of consumer preference tending towards the mode of rehabilitation they received mirrors those outlined by Naylor et al. [24] in a previous study investigating patient preferences after knee arthroplasty in the public sector. That preference is linked to past experience suggests a general satisfaction with therapy received. It is unclear whether this is because the mode of rehabilitation delivery undertaken by each patient was the most appropriate for their unique situation, or that the perceived quality of care across modes was high. If the latter, this does not discount the importance of the mode itself, but suggests that alternative modes of rehabilitation are likely to be acceptable to patients if they are of high quality. As outlined by Perkovic et al. [29], if it can be demonstrated that alternative models of care are as effective as existing services, but cheaper, an efficient system would encourage the uptake of these services.

A recent randomised, controlled trial, published after this study concluded, indicated that inpatient rehabilitation was not more effective than a monitored home-based program when measured across a range of outcomes up to 52 weeks post-surgery among adults undergoing uncomplicated total knee arthroplasty [30]. These findings do not support inpatient rehabilitation for this group of patients. With these findings in mind, one point for consideration for healthcare providers, policy makers and insurers is a re-examination of the pathway to inpatient rehabilitation, to look for ways to reserve this intensive, and costly, mode for patients who have a specific clinical or social need for it. Our interviews showed that healthcare staff involved in these decisions, particularly OS, are aware of alternatives, and often have a preference for them. However, they are reluctant to modify consumer expectations and sense of entitlement, or simply do not see the need for this course of action. This principle of entitlement alluded to by both consumers and clinicians during the interviews can be seen to be somewhat at odds with the attribute of efficiency, which has long been a pillar of the Australian healthcare system [31]. This is particularly pertinent when a sense of entitlement overrides clinical judgement, as inappropriate care is inefficient.

When exploring possible reasons for this situation, a number of potential dilemmas may be responsible. One reason may be the additional time it would take within an orthopaedic consultation to provide information and talk through other options available; a review of 38 studies looking at the most frequently reported barrier to implementing shared decision making in clinical practice was time constraints [32]. Another may be the financial implications for surgeons who did not refer patients with a preference for inpatient rehabilitation to that mode. If a patient presented with a strong preference for inpatient rehabilitation that the clinician opposed, the patient could always go to another surgeon who supported or allowed their preferred treatment. The formulation of guidelines or standards for orthopaedic surgeons or other healthcare providers regarding the reservation of inpatient rehabilitation for patients with a specific clinical or social need might be considered to counter this. OS and healthcare providers need to also consider these needs prior to surgery, and play a more active role in promoting modes of rehabilitation that would address these. Private health funds could do the same for their members by looking to address competing financial tensions, and develop supplemental services and clearer pathways to alternative rehabilitation modes and settings. One option may be the removal of financial barriers in the form of ‘out of pocket expenses’ for members who elect to go directly home from the acute facility after arthroplasty. Also, although familial relationships cannot be changed, what could be offered to facilitate an early discharge home after surgery are services such as transport, cleaning and meal preparation, which were identified as determining factors for participants who chose not go to inpatient rehabilitation, and from those to whom alternative modes were preferred. Another avenue which could be considered to at least partially overcome the issue of transport is domiciliary physiotherapy.

A separate factor influencing the treatment pathway after arthroplasty may be the business model of the site in question. The site at which inpatient rehabilitation was generally presented as ‘part of the package’ was run by a healthcare organisation which owned both the acute and rehabilitation facilities. The second, where patients went through surgery and associated treatment without knowledge of inpatient rehabilitation as an option, was owned by an organisation which did not have a business interest in any local rehabilitation facilities. Further study in this area may establish whether this pattern exists on a wider scale.

The clear preference for inpatient rehabilitation by RS is largely unsurprising and revolved primarily around the perceived clinical benefits of this mode. The fact that these same RS benefit personally from the provision of this service was acknowledged during the interviews. Their preferences in this regard, however, seemed to have limited influence on the decision to send patients to inpatient rehabilitation, as they were generally not a part of the decision pathway leading to that setting.

## Conclusion

This study has provided a unique opportunity to articulate stakeholder attitudes towards various rehabilitation types, and the factors which guide consumer and clinician decisions relating to the utilisation of different rehabilitation modes following arthroplasty. An understanding of consumer and clinician preferences for rehabilitation, particularly inpatient rehabilitation, should help to inform ongoing and future models of care delivery, hand-in-hand with new evidence of effectiveness as it emerges. This will be particularly crucial to consider if alternative, less costly models of care are to be developed for, and acceptable to, the private sector.

This study indicates that no one mode of rehabilitation provided following knee or hip arthroplasty is singularly preferred by stakeholders. Consumers may have a preference for the more expensive rehabilitation approaches, but if such modes are shown not to be more effective, then there is a need to explore less costly modes of rehabilitation following arthroplasty. If change is to be enacted, clinicians involved in the decision-making process will need to consider the comparative effectiveness of rehabilitation types, as well as the clinical and social needs of their patients, and play a more active role in promoting appropriate options to their patients

## Additional file

Additional file 1: Appendix 1; Appendix 2; Appendix 3. Interview templates. (DOCX 17 kb)

## Abbreviations

EQ5D: EuroQol five dimensions questionnaire
OS: Orthopaedic surgeon
PT: Physiotherapist
RCT: Randomised controlled trial
RS: Rehabilitation specialist
THA: Total hip arthroplasty
TKA: Total knee arthroplasty
VAS: Visual analogue scale
WOMAC: Western Ontario and McMaster Universities Osteoarthritis Index
