# Supplementary material for: Understanding consumer and clinician preferences and decision making for rehabilitation following arthroplasty in the private sector
Source: BMC Health Serv Res. 2017 Jun 19;17:415. doi: 10.1186/s12913-017-2379-9 (PMC5477339; doi:10.1186/s12913-017-2379-9)
Supplement: Additional file 1: — Appendix 1; Appendix 2; Appendix 3. Interview templates. (DOCX 17 kb) [file 12913_2017_2379_MOESM1_ESM.docx]

**Additional file1: Appendix 1 - Patient interview script**

1. Why did you choose to go to inpatient rehabilitation after your surgery?
2. Did you see any other benefits of inpatient rehabilitation?
3. Were there any other factors that influenced this decision?
4. Did you talk to anyone about the decision?
5. Was there anyone who influenced your decision?
6. Did you have any carer support and, if so, how much and in what form?
7. Did you have any other social support (neighbours, community, etc.) If so, how much and in what form?
8. Where you presented with any alternatives regarding your therapy post-surgery?
9. I will now present you with five options for rehabilitation after surgery. Which of these holds the most appeal, and why?
   1. Outpatient group therapy: Pt goes to the hospital at 2, 4 and 8 weeks post discharge for a group-based outpatient physiotherapy session, where support is provided and exercises upgraded.
      1. Transport to and from visits is provided
      2. Transport to and from GP is provided
   2. Outpatient one-to-one therapy: Pt goes to the hospital twice a week for 6 weeks for a 1:1 outpatient physiotherapy visit, where support is provided and exercises upgraded.
      1. Transport to and from visits is provided
      2. Transport to and from GP is provided
   3. Domiciliary therapy: A physiotherapist comes to pts home twice a week for three weeks to provide support and upgrade exercises. For two weeks, meals are provided every day (lite-and-easy style) and a cleaner comes in once a week.
   4. Hotel-based rehabilitation: Pt are transferred to a local 4 star hotel for 2 weeks where all meals are provided, along with daily 1:1 and/or group based therapy
   5. Inpatient rehabilitation: Pt is transferred to a local rehabilitation facility for 2 weeks, where they receive daily 1:1 or group based therapy.
10. Can you place the remaining four in order of preference?

**Additional file 1: Appendix 2 - Carer interview script**

1. What is your relationship to X?
2. What support did you provide to X?
3. What form did this support take? Prompts:
   1. transport
   2. meals
   3. ADL
   4. Emotional
   5. motivational
4. What was your opinion of the rehabilitation X received after their surgery?
5. I will now present you with five options for rehabilitation after surgery. Which of these holds the most appeal, and why?
   1. Outpatient group therapy: Pt goes to the hospital at 2, 4 and 8 weeks post discharge for a group-based outpatient physiotherapy session, where support is provided and exercises upgraded.
      1. Transport to and from visits is provided
      2. Transport to and from GP is provided
   2. Outpatient one-to-one therapy: Pt goes to the hospital twice a week for 6 weeks for a 1:1 outpatient physiotherapy visit, where support is provided and exercises upgraded.
      1. Transport to and from visits is provided
      2. Transport to and from GP is provided
   3. Domiciliary therapy: A physiotherapist comes to pts home twice a week for three weeks to provide support and upgrade exercises. For two weeks, meals are provided every day (lite-and-easy style) and a cleaner comes in once a week.
   4. Hotel-based rehabilitation: Pt are transferred to a local 4 star hotel for 2 weeks where all meals are provided, along with daily 1:1 and/or group based therapy
   5. Inpatient rehabilitation: Pt is transferred to a local rehabilitation facility for 2 weeks, where they receive daily 1:1 or group based therapy.
6. Can you place the remaining four in order of preference?

**Additional file 1: Appendix 3 – Clinician interview script**

1. What do you see as the benefits (if any) of inpatient rehabilitation after knee and hip joint arthroplasty?
2. Do you see any downsides to patients attending inpatient rehabilitation after surgery?
3. What would lead you to recommend inpatient rehabilitation for a patient following surgery?
4. Are there any other factors that would influenced this decision?
5. Would you talk to anyone to help make this decision?
6. What are the current treatment alternatives available for these patients in your area?
7. Can you think of any other options that should be considered as treatment options following knee or hip arthroplasty?
8. I will now present you with five alternatives for rehabilitation after surgery, which I will ask you to rate (Likert scale used).
9. What are the possible positive and negative components, if any, of each?
   - Outpatient group therapy: Pt goes to the hospital at 2, 4 and 8 weeks post discharge for a group-based outpatient physiotherapy session, where support is provided and exercises upgraded.
     - Transport to and from visits is provided
     - Transport to and from GP is provided
   - Outpatient one-to-one therapy: Pt goes to the hospital twice a week for 6 weeks for a 1:1 outpatient physiotherapy visit, where support is provided and exercises upgraded.
     - Transport to and from visits is provided
     - Transport to and from GP is provided
   - Domiciliary therapy: A physiotherapist comes to pts home twice a week for three weeks to provide support and upgrade exercises. For two weeks, meals are provided every day (lite-and-easy style) and a cleaner comes in once a week.
   - Hotel-based rehabilitation: Pt are transferred to a local 4 star hotel for 2 weeks where all meals are provided, along with daily 1:1 and/or group based therapy
   - Inpatient rehabilitation: Pt is transferred to a local rehabilitation facility for 2 weeks, where they receive daily 1:1 or group based therapy.
